# Caregiver, Parent with At‐Risk Children

**DOI:** 10.1002/alz.086678

**Published:** 2025-01-03

**Authors:** Tanya Steel

**Affiliations:** ^1^ Cure MAPT FTD, Sarasota, FL USA

## Abstract

My beloved husband, Robert Steel, died at 58 from MAPT FTD, a disease that stretches back through his family’s generations. Before we married he told me his father died of early‐onset Alzheimers and he was terrified he would inherit that disease. In his early 50s he began exhihibiting symptoms that I attributed to stress–his work as a respected high school teacher began to slip, he began to lose the ability to have a visual map in his head, he began to repeat stories, his behavior became more compulsive, forgot how he was related to a favorite cousin. He had to leave the teaching profession and I had to support our family, with my identical twins soon headed to college.

I took him to a reputed neurologist who said perhaps he was depressed. This diagnosis was repeated with other dementia specialists. One day he got into a car accident and left the scene, something that was the opposite of his character. From that day he seemed in a fog and almost unreachable. It was then that I got him diagnosed by Columbia Presbyterian’s Dr. Karen Marder, an FTD specialist. His Behavorial Variant FTD caused by the MAPT gene has likely affected many generations before him, many of whom died fairly young and often of mysterious circumstances.

Since that time, I have led a support group for AFTD, begun a book for caregivers of dementia patients, and become a founding member of Cure MAPT FTD. With my three decades of experience on television, panels, public speaking including four speeches at The White House with Mrs. Michelle Obama alongside, I am committed to using my communication skills to help cure this insidious disease. I will speak from the caregiver’s vantage point, discussing the difficulty FTD specifically presents for caregivers and patients–from finances to safety–what clinicians can do to better support both constituents, the frustration in the lack of MAPT‐specific trials, the importance in spreading awareness to thus help others gain quicker diagnosis, and the decision to not yet test my sons.

